# Rethinking Plant Proteins: Innovations in Nutrition, Processing, and Food Development

**DOI:** 10.1007/s11130-026-01509-w

**Published:** 2026-05-11

**Authors:** Nishant Kumar, Nutan Kaushik, Pratibha, Rushali Jaiswal, Clara Mariana Gonçalves Lima, Anka Trajkovska Petkoska, Marek Kieliszek

**Affiliations:** 1 Amity Food and Agriculture Foundation, Amity University, Sector 125, Noida, Uttar Pradesh 201301 India; 2 Center of Management and Humanities (CMH), Punjab Engineering College (Deemed to Be University), Sector 12, Chandigarh, 160012 India; 3 Department of FST, National Institute of Food Technology Entrepreneurship and Management, Kundli, Sonepat, 131028 India; 4 Department of Biological Chemistry, Regional University of Cariri, Ceará, 1161 Brazil; 5 Faculty of Technology and Technical Sciences -Veles, University “St. Kliment Ohridski”– Bitola, Dimitar Vlahov 57, 1400 Veles, Republic of North Macedonia; 6 Department of Food Biotechnology and Microbiology, Institute of Food Sciences, Warsaw University of Life Sciences—SGGW, Nowoursynowska 159 C, 02-776 Warsaw, Poland

**Keywords:** Plant proteins, Meat alternative, Structure, Proteins extraction, Protein modification, Allergenicity

## Abstract

Plant-based proteins are novel nutrient sources that are widely available in nature and cost-effective to produce compared to animal proteins, while contributing to lower greenhouse gas emissions and reducing risks to both environmental and human health. Thus, the present work comprehensively reviews plant-based proteins as an alternative to conventional / animal proteins. The review article highlights why plant proteins should be considered a novel nutrient source and an alternative to animal proteins. Various plant protein-derived sources and their corresponding extraction methods, along with their associated limitations, are reviewed. In addition, the review article highlights plant protein modification methods, interfacial behavior, protein structure, interactions, and functional properties. The effects of different physical and environmental conditions and factors on the functional and nutritional properties of plant proteins are also discussed. In addition, the challenges associated with plant proteins, such as allergenicity, consumer acceptance and perceptions, food safety, regulatory issues, and technological challenges, are also explored.

## Introduction

Food security is one of the most critical challenges, particularly amid rapid population growth. The population is projected to reach approximately 9.7 billion by 2050. Consequently, demand for food and nutrition is increasing rapidly to maintain human health [30]. Proteins are an essential nutrient required for human consumption (0.8 g/kg of body weight) daily for enzymatic synthesis, hormone production, and the repair of body tissue. Proteins can be derived from various conventional animal-based sources (including meat, dairy, eggs, and fish products) as well as plant sources such as cereals, nuts, fruits, and vegetables. The quality of proteins depends on amino acid profiles, purity, bioavailability, accessibility, and digestibility [71]. In the last decade, consumers have become more aware of their health and their daily diets to maintain good health and immune function. The proteins market size is expected to reach 27.48 billion US dollars in 2034, up from 13.18 billion US dollars in 2025 [55].

Animal-based proteins provide essential amino acids vital for human metabolism but face significant challenges due to rising demand and environmental impacts. Livestock production occupies nearly 77% of agricultural land, consumes 30% of freshwater, and contributes 12–20% of anthropogenic greenhouse gas emissions [83]. Furthermore, concerns regarding zoonotic disease transmission [51] have shifted consumer preference toward sustainable, eco-friendly alternatives. Emerging plant-based proteins and meat analogs offer a viable solution to mitigate these ecological and economic pressures while addressing technological obstacles in the food industry.

According to the Food and Agriculture Organization (FAO) [53] and the World Health Organization (WHO) [84], 2019, sustainable diets must be healthy, safe, and nutritionally adequate with minimal environmental impacts. Reflecting this shift, the plant-based protein market—valued at $23.89 billion in 2025—is projected to reach $34.97 billion by 2030, growing at a compound annual growth rate (CAGR) of 7.9%. The growing market for plant proteins is driven by a rapid shift in consumer choice away from animal proteins toward vegan, flexitarian, and vegetarian options [54]. Based on these trends, the Generation Z population is projected to shift from animal to plant-based proteins. Plant-based proteins are a cost-effective alternative to animal protein production, offering higher concentrations of bioactive compounds and greater accessibility. The consumption of plant-based proteins may reduce the risk of certain health conditions, such as cholesterol and high blood pressure issues, improve obesity-induced metabolic dysfunctions, and prevent cardiovascular and carcinogenic diseases [51]. There is minimal in-depth literature on plant-based proteins as alternatives for food applications, and challenges remain. Therefore, this review aims to investigate the potential of plant proteins as substitutes for animal proteins. In addition, this article discusses the effects of various factors (physical, environmental, processing, and extraction methods) on protein quality and purity, along with their applications and challenges. Moreover, the article highlights the structure of plant proteins, mechanisms of interfacial behavior, plant protein interactions, and functional properties, as well as technological and regulatory challenges.

### Plant-Based Proteins are a Novel Source of Nutrients

Plant-based proteins are considered nutritionally rich for the human diet and account for 49% of the supply, without depleting natural resources such as agricultural land and water, and without increasing greenhouse gas emissions. There has been an increased demand and consumption of plant-based protein alternatives to conventional and animal proteins (red meat, dairy products, seafood, eggs), with a focus on minimizing environmental degradation and human health risks [77]. Plant-based proteins are considered a sustainable and healthy option for commercial food applications.

### Plant-Based Proteins Sources

**Fig. 1:**
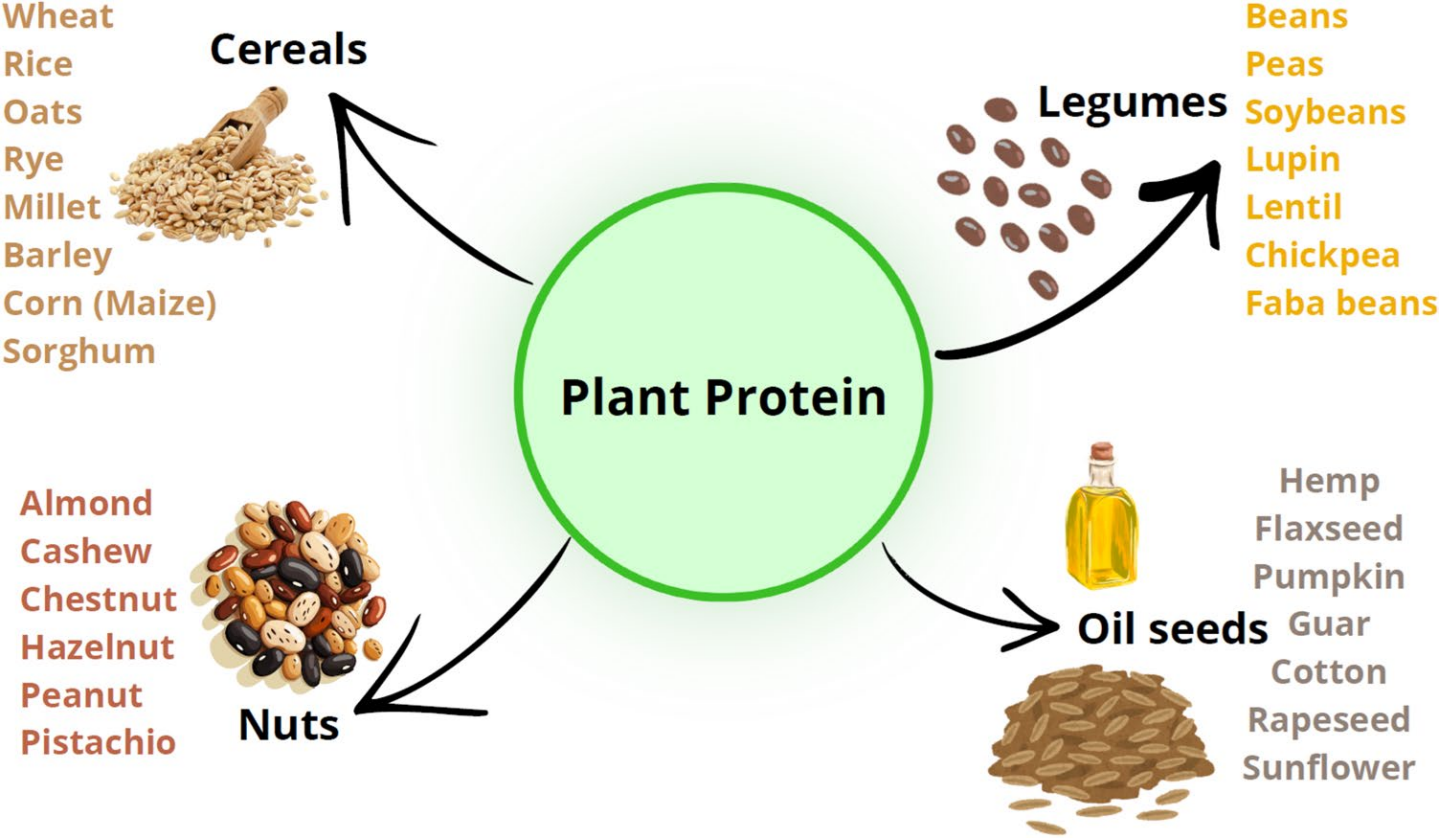
Different plant-derived sources for the sustainable production of plant proteins

The plant sources such as oil seeds (pumpkin, cotton, sesame, sunflower, rapeseed flaxseed, karaya seed), legumes (faba beans, beans, peas, soybeans, lentil, lupin, chickpea), edible seeds (buckwheat, quinoa), cereals (corn, wheat, barley, sorghum, oats, rice, maize, rye), pseudocereals (chia seed, amaranth), nuts (cashew, almond, peanuts, pistachio) and some tuber crops, fruits & vegetable, and by-products and herbs such as Quelite are generally considered good source for plant proteins [7, 36] (Fig. 1).

However, plant-based proteins are more abundant in nature and cost-effective to produce compared to animal proteins. Nevertheless, plant proteins are an important component of human diets; their nutritional quality may be constrained by factors such as reduced digestibility and bioavailability, limitations in essential amino acid composition, the presence of antinutritional compounds, and less favorable sensory attributes. Consequently, only 3% of plant proteins were converted into animal proteins, indicating the inefficiency of the process [73]. Therefore, plant-based proteins can be utilized to develop functional food such as meat analogs, alternatives of dairy products (milks, yogurt, cheese), snack products, bars, baked products, and fortified beverages after extraction and isolation from plant sources such as legumes, oilseeds, cereals, edible seeds, pseudocereals, nuts, etc.

Several methods and techniques, such as electrospinning, extrusion, blending with hydrocolloids, freeze structuring, shear cell technology, 3D printing, and wet spinning, have been used to structure meat analogs as alternatives to meat [17]. Previously, various seed flours were used seed meals such as gum karaya, pepper seed, lacquer seed, pennycress seed, *Zanthoxylum* seed, hemp seed, mustard seed, coffee seed, cotton seed, rapeseed, flax seed, tomato seed, pennycress, chia seed, *Camellia oleifera* seed meal, copra meal, and soybean seed, *Acer truncatum* seed, sesame seed, bitter gourd, perilla seed, *Gossypium* seed, sunflower, olive, rapeseed, lupin seed, Pumpkin, pomegranate and grape seed, soybean seed, chickpea, flixweed seed, peanuts, pumpkin, sunflower and soybean seeds, *Xanthoceras sorbifolia* seed, cumin seed, quince seed, moringa seed, orange seed, grape seed, *Silphium integrifolium* seed, jatropha and soybean seed, camelina, flixweed seed, and amaranth seed for the applications in the food processing sector. They have extracted proteins from seed oils using various conventional and emerging extraction methods.

Isolates (> 90%) and concentrates (50 - 70%) are two forms of proteins defined by yield and quality after extraction. Proteins are categorized into globulins, albumins, prolamins, and glutenins based on quality [22]. Each protein class has its own properties, including solubilization medium and pH. For example, globulins, glutelins, and prolamins are soluble in salt, alkali, and alcoholic media, whereas albumins are soluble in aqueous media and susceptible to heat coagulation. In this case, both albumin and globulins are considered major proteins essential to human health, as they regulate body functions and support healing and tissue growth. Albumin proteins are generally soluble in aqueous media, are globular, and coagulate upon heating. Globulin proteins are soluble in salt solutions. Prolamin proteins dissolve in alcohol/water mixtures. Globulin proteins have a globular structure and a higher molecular weight than albumins. Glutelin proteins are soluble in alkaline medium and contain intermolecular disulfide bonds [37].

### Structure of Plant Proteins

The structure of plant proteins mainly depends on their amino acid components, peptide subunits, and their soluble and denaturation properties. The properties of plant proteins varied depending on the type and source of the proteins. In the food processing industry, protein isolates from soy, pea, peanut, wheat, and sunflower are widely used compared to other plant proteins. Protein structures are categorized into four types (primary, secondary, tertiary, and quaternary (complex)) based on the organization level of amino acids and the types of interactions that stabilize them. The primary structure of proteins is composed of linear chains of amino acids [4]. The secondary structure of proteins also comprises amino acids, which are stabilized by hydrogen bonds (forming alpha helices and beta pleated sheets) within the polypeptide. Moreover, the tertiary structure of proteins is a three-dimensional structure determined by amino acid side chains along the polypeptide backbone, and the quaternary structure influences the three-dimensional shape of proteins via side-chain interactions between two or more polypeptides [65].

### Extraction and Isolation of Proteins from Plant Sources

The extraction and isolation of proteins from plant sources are crucial steps to obtain proteins from different types of plant sources, such as legumes, oilseeds, cereals, edible seeds, pseudocereals, nuts, etc., with a higher yield of proteins along with the good functional quality of proteins, which may depend on the types of sources and extraction methods [2]. The extraction process of proteins from plant sources involves various steps, such as defatting, extraction, precipitation, and purification, to isolate the desired proteins by removing other nutritional components, including fat, fiber, starch, and other impurities in the samples [8]. Several conventional and emerging techniques such as alkaline extraction, sub and supercritical water extraction, membrane ultrafiltration, deep eutectic solvent extraction, reverse micelles extraction, pressurized, high voltage electrical discharge, enzymatic assisted extraction, microwave assisted extraction, high pressure assisted extraction, pulse electric extraction methods etc. used for the extraction and isolation of proteins isolates and concentrates from the plant sources [51]. The recent novel extraction methods are generally considered superior to conventional methods, as they yield higher protein yields. These methods also produce higher-quality proteins with better functional properties. This might be attributed to the breakdown of the cell wall, composed of complex polysaccharides, which provides support for proteins by retaining them intracellularly, thereby influencing protein quality [47]. Previously, various researchers have reviewed comparisons between conventional and nonconventional extraction methods and their effects on protein quality and functionality [45]. Moreover, previous researchers have used various extraction methods to isolate proteins from plant sources, such as almond kernel, moringa seed, rice, and sunflower seed [67]. However, the recovery of proteins from plant sources may also depend on factors such as plant type, cultivar, agroclimatic conditions, and extraction method. Each conventional and nonconventional extraction process has its advantages and limitations for extracting proteins from plant sources [56]. Table 1 presents the advantages and disadvantages of various extraction methods for plant protein extraction.

**Table 1:** Extraction methods and limitations of plant proteins

Types of extraction methods	Key findings	Disadvantage	References
Alkaline extraction (Conventional)	Easy to process and handle Cost effective Higher extraction yield	Long processing Produced acid/alkaline water, which causes environmental pollution Low-quality protein with less functionality	[81]
Supercritical water extraction	Eco-friendly due to the use of organic solvents Short extraction time duration Suitable for commercial scale, extraction of high-quality protein with good physico-chemical and functional properties	Degradation and hydrolysis of protein into small fragments and subsequently free amino acids due to high pressure and temperature Denaturation of proteins High-cost equipment	[50] [82]
Membrane ultra-filtration	Suitable for separating high molecular weight proteins Improve structural and functional properties by controlling operational parameters Non-toxic/eco-friendly Less denaturation of protein Separate proteins based on molecular weight Higher extraction yield of protein Improving the digestibility and other functional properties of protein	Required high energy High cost Affected amino acid properties with higher lipophilicity Chances of contamination	[81]
Pasteurized liquid extraction	Eco-friendly and non-toxic in nature Automatic extraction method High protein yield Shorter extraction time	Chances of denaturation and racemization of protein Affected structural changes due to a mixture of both L and D forms of amino acids Higher cost of equipment Difficult in the optimization process of parameters	[24, 26]
Deep eutectic solvent extraction	Biodegradable, low-volatile solvents Low pressure (vapor) and non-toxic Non-inflammable Higher protein extraction Higher solubility and other functional properties of protein	Chances of dissolution of protein due to higher viscosity Affects the heat and solvent mass transfer rate Lower extraction coherence Some deep eutectic solvents showed mild toxicity	[26]
Reverse micelles extraction	Easy to modify the process.' Low cost Recovery of nonpolar solvent and surfactants Scaling up technology Less denaturation of protein	Consumption of high amounts of water and energy Lack of extraction efficiency Loss of solvents and surfactants due to the insoluble complex of protein Low purity of protein	[64]
High-voltage electrical discharge	Less environmental impact Higher efficiency of cell destruction Recovery of a high yield of protein Low consumption of solvent Required less temperature for operating Less chance of denaturation of protein and maintained higher functional and structural properties	May affect functional and structural properties due to oxidation of proteins Use at pilot scale only	[42]
Ultrasound-assisted extraction	Eco friendly Non-toxic Less denaturation of protein Easy to operate Increased extraction yield and properties Improved functional properties of protein	Higher consumption of energy and organic solvents in the samples Chance for denaturation of protein at high pH Use at pilot scale only May affect functional and structural properties due to oxidation of proteins	[25, 52]
Pulse electric field extraction	Eco-friendly-inflammable organic solvents Low consumption of energy Less time for extraction Effective in improving the protein solubility and digestibility Higher extraction yield	Operational issue with the formation of bubbles during the process Non-uniform treatment Expensiveness of the instrument Commercial units are not available	[6]
Enzymatic-assisted extraction	Increased extraction yield of protein Minimize the use of the extracting solvent Non-toxic Quick extraction process Can be used at a commercial scale	Costly process due to the higher cost Needs to optimize the process, including the number of enzymes, for higher recovery of protein from plant sources Variations in enzymes (purity, efficiency, and catalytic activity)	[68]
Microwave-assisted extraction	Easy to handle Required a low volume of solvents Less emission of carbon dioxide Improved the protein solubility and digestibility	High cost of equipment The chance of oxidation of protein, which may affect the protein properties due to dielectric absorption Required a longer time for the extraction process	[25, 68]
High-pressure assisted extraction	Eco-friendly approach Improve mass transfer Enhance extraction efficiency Reduced extraction time and consumption of solvents Improved functional properties of protein isolates Lower thermal degradation	Expensiveness of the instrument Consumption of higher energy Production of byproducts Chance of structural alteration in protein due to breakdown of hydrophobic interaction and salt bridge	[15]

### Plant Proteins Modification Methods

Plant-based proteins offer a way to mitigate the negative environmental impacts by reducing greenhouse gas emissions. Besides, the several advantages of plant proteins, such as health benefits and eco-friendliness, face numerous challenges in the food processing industry due to inherent properties, including lower solubility, protein quality, foaming and emulsifying properties, rheological properties, fat and water absorption, and protein allergenicity. However, to use plant-based proteins in food formulations, modifications are required to improve their functionality. These functional properties are significant for food formulations that offer existing health benefits. To improve the properties and functionality of plant proteins, various modification methods - both traditional and innovative - are used to enhance their technological usefulness [72]. The use of traditional protein extraction and modification technologies, such as alkaline extraction, has several limitations due to their greater resource consumption. In addition, the denaturation of protein structure is not easily accelerated by traditional methods, which may also negatively affect the functional properties of plant proteins, including digestibility and bioactive compounds, as well as the environment by releasing toxic byproducts. To overcome the limitations of traditional plant protein extraction methods, innovative, eco-friendly processing methods can be used to enhance plant protein functionality. The novel methods of plant proteins modifications such as thermal (high pressure processing, supercritical, micro-fluidization, ultrasounds, microwave, radiofrequency heating) and non-thermal (non-thermal plasma, ozone, ultraviolet, ionizing radiation) are potential to improving the proteins quality and functional properties such as nutritional availability, foaming, emulsifying, fat & water absorption ability, viscosity, reduced plant proteins allergens and antinutrient factors by altered the proteins structure (primary, secondary, tertiary and quaternary) by disintegrated the interaction and bonding between the proteins molecules [74]. A recent study by Rout et al. [59] investigated the effects of various modification methods on protein quality and functional properties, as well as the mechanisms underlying these effects. Previously serval researchers have applied various novel proteins modifications techniques such as ultrasonication, ultrasound, supercritical fluid extraction, microwave heating, pulsed electric field [10] and ozone treatments to improve the quality as well as the functional and structural properties of plant based proteins such as apple seed proteins, radish seed proteins, rice bran proteins, lentil proteins, pea proteins, soybean and chickpea proteins, respectively. Moreover, microbial fermentation is primarily used to modify plant proteins by altering their structure and composition, and to enhance functional properties such as solubility, digestibility, emulsifying properties, and other protein quality attributes. Various microorganisms, such as *Lactobacillus*, *Enterococcus*, *Leuconostoc*, *Pediococcus*, and other microbial species, have been used in the fermentation of plant proteins to improve the nutritional and functional quality of proteins by breaking down the complex compounds and synthesizing the complex of vitamins and other growth factors due to catabolic and anabolic properties of microorganisms.

Furthermore, microbial fermentation also helps alter the composition of antinutrients, such as phytate, tannins, and oxalates, in plant proteins, thereby directly influencing their functional properties and digestibility. It facilitates the release of bioactive compounds by breaking down cell walls [19]. Previous studies have demonstrated that fermentation significantly improves the functional and nutritional properties of plant proteins [13].

### Mechanism of Interfacial Behavior of Plant Proteins

Plant proteins have garnered interest as an alternative to animal proteins for stabilizing emulsions and foams due to the potential formation of a strong relation between cohesive layers and interaction between proteins-proteins (after adsorption) and the amphiphilic nature of the plant proteins, which can stabilize the interfaces [63, 76]. Plant protein alternatives to conventional and animal sources are a sustainable approach to overcoming food security and environmental challenges. Previous researchers have confirmed that plant proteins contain various nutritional components, including amino acids, as well as solubility, gelling, emulsifying, and other functional properties. Moreover, modification of plant proteins also influenced their techno-functional properties and altered the amino acid composition [76]. Proteins derived from plant sources are generally lower in hydrophobicity, emulsifying, and foaming properties than animal-based proteins due to slower absorption rates. It is worth noting that their functionality may also be affected by the extraction process [49]. This could result from weaker in-plane interactions and a decrease in the interfacial film's stiffness. The protein extraction procedure may result in significant globulin aggregation, which lowers solubility. Several processes can cause irreversible protein aggregation. Still, two crucial ones are protein precipitation at the globulin's isoelectric point (pI) and interaction between phenol and proteins during the alkaline extraction process.

It is worth noting that increased protein aggregation after precipitation leads to reduced solubility and weakened functional properties, such as emulsification and interfacial film formation (proteins stabilize the oil–water interface) [28]. Additionally, phenols may oxidize during alkali extraction, which is sometimes carried out at pH values as high as 13. Highly reactive quinones that can covalently bind to proteins and form large aggregates can be formed by oxidized phenols [33]. Plant-protein functionality can be enhanced by altering proteins' physicochemical properties or by using gentler extraction techniques. Optimizing the extraction process is one way to increase the solubility of the globulin fraction, while preserving the albumins (either as a distinct stream or in combination with globulins). Albumins and globulins can be extracted together by avoiding the protein precipitation stage. Small non-protein components, such as phenols, carbohydrates, and minerals, can be removed from this protein mixture through diafiltration [48]. This technique is referred to as a mild extraction approach. Another emerging technique that produces protein-rich extracts with a substantial portion of their original protein structure intact is dry fractionation combined with air classification [43]. Higher protein yields and lower resource requirements are two additional significant advantages of mild purification [38]. For proteins to be adequate in terms of interface, foam, and emulsion stability, it is essential to preserve the native protein structure as much as possible. Avoiding an extremely alkaline pH to prevent phenols from oxidizing and skipping the protein precipitation stage will help prevent process-related protein aggregation. It is essential to thoroughly investigate the precise effects of these possibly aggregate-inducing events on plant-based proteins [63]. Interfacial rheology and synchrotron radiation techniques are two methods for studying interfacial properties, which are substantially determined by the protein structure at the interface. Although simulating a large number of protein molecules adsorbed at a fluid–fluid interface remains infeasible, atomistic molecular dynamics simulations can also provide additional insight into the specifics of the interfacial structure [16]. Perhaps beneficial details about the interfacial microstructure can already be obtained from coarse-grained simulations of smaller structures (such as short peptides rich in either α-helix or β-sheet structure). This method of simulating simpler molecules, such as block copolymers, revealed a rich in-plane phase behavior that depended on molecular features and the strength of molecular interactions [63]. Determining the best modification approach for plant-based proteins and creating interfaces with functional features would be made possible by further investigation into the relationship between protein microstructure at the interface and interfacial (mechanical) properties.

### Factors Affecting Plant Protein Properties

**Fig. 2:**
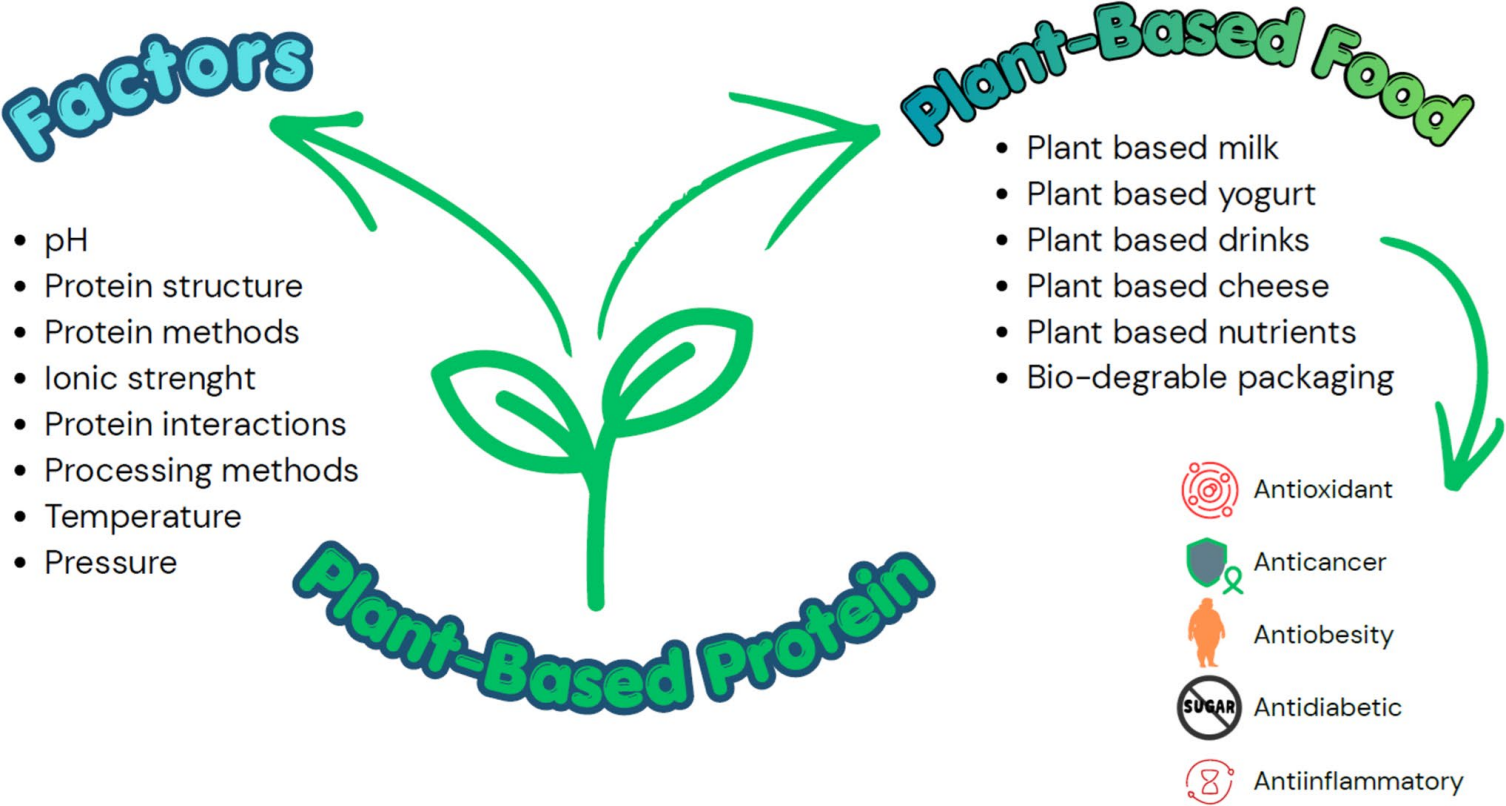
Factors responsible for affecting the quality of protein and applications of plant-based protein in foods

The quality and functional properties such as solubility, yield, amino acids and their nutritional value, digestibility, foaming and gelling property and other properties of plant-based proteins may be affected by several physical and environmental conditions (solvent, time, pH, ionic strength, pressure, temperature), processing methods and extraction process (cooking, boiling, germination, irradiation, homogenization etc.) and structure of proteins, etc. Figure 2 illustrates the factors that influence the quality and functionality of plant proteins during extraction and isolation [35]. Several researchers have investigated and reported on how factors significantly affect the quality and functionality of proteins. For example, Sá et al. [60] reviewed and highlighted emerging technologies such as ultrasound, ultrasonication, microwave, supercritical fluid extraction, high-pressure homogenization, ohmic heating, enzymatic, and cold plasma methods for the extraction of plant proteins, which exhibit excellent functional and nutritional properties due to conformational changes. Additionally, Szepe et al. [70] have reviewed and reported on the environmental and genetic factors influencing protein quality and techno-functionality. The processing methods, such as cooking, germination, autoclaving, irradiation, microwave, freeze & spray drying, fermentation, and extrusion process generally improved the plant proteins quality by deactivating the antinutritional factors, which influenced the improvement in nutritional and functional properties, such as digestibility, and to enhance their application in food products [32]. Moreover, Sa et al. [61] investigated the effects of various extraction methods, including conventional thermal, microwave, and ultrasound treatments, on the functional properties and quality of pumpkin seed meal proteins. They have reported the operational parameters, and their conditions may significantly affect the proteins. Additionally, the use of emerging methods helped minimize antinutritional factors in proteins and improve their digestibility.

### Proteins Interactions and Functional Properties

The activity of proteins is generally determined by their interactions with other proteins, polysaccharides, lipids, polyphenols, and various other molecules. Protein interactions can occur with the other copy of the same protein or with distinct proteins, forming homo- and heterooligomers, respectively. The protein–protein interaction network, known as the interactome, is influenced by differences in protein association, which can significantly alter interactions through biological factors. Various factors significantly influence the interfacial behavior and protein–protein interactions, including pH, extraction methods, ionic strength, time, temperature, and processing and modification techniques such as pH alterations, ultrasonication, microwave assisted extraction, etc. due to which there is conformational unfolding of protein structure [78]. Recent studies show that shifting to an alkaline pH increases protein solubility by boosting electrostatic repulsion, as reported by Chen et al. [11] for rapeseed protein isolate. This change helps proteins adsorb quickly at the oil–water interface. Moreover, studies on ultrasound-assisted extraction indicate that acoustic cavitation decreases molecular weight and helps develop elasticity at the interface film [80]. Similarly, microwave-assisted extraction (MAE) causes quick dipole rotation. This rotation helps unfold globular structures and increases the exposure of buried hydrophobic areas [14]. However, the proteins' interaction with the other components, such as polysaccharides, lipids, and polyphenols, may affect the behavior and functions of proteins during the manufacturing, processing, storage, and addition of other ingredients in food formulations, which directly affect the functional properties, such as rheological, gelation, solubility, and film-forming ability of proteins [57]. Generally, polyphenol compounds do not directly interact with other micronutrients in plant cell vacuoles. The interaction between polyphenols and cellular molecules (intra- and extracellular) occurs during food processing, as plant tissue is disrupted. Which process influenced the oxidation of polyphenol compounds and simultaneously interacted with bound phenolic groups (the side chains of proteins) [23]. Several researchers have investigated the effects of protein–polyphenol interactions and shown significant changes [67]. For example, Alu’datt et al. [3] reported that 56 - 62% of phenolic interaction with the flaxseed protein isolates altered their functional properties. The sunflower protein isolates exhibited decreased protein band intensity, increased hydrophobicity, and higher denaturation temperatures due to phenolic interactions. Furthermore, the interaction between plant proteins and polyphenols was found to enhance the solubility, excellent foaming and emulsifying properties, thermal stability, and other properties of *Cinnamomum camphora* seed protein isolates [75].

### Challenges in the Application of Plant Proteins in the Food Industry

With increasing demand for plant-based and alternative proteins as substitutes for animal-based proteins, there is a need for a sustainable solution that provides food security while offering environmental benefits through the responsible use of natural resources. Alternative proteins could be a potential way to reduce calorie intake and improve human bone health. Consequently, the nutritional and functional values of plant proteins and food products may be affected by the presence of various antinutrients and allergenic compounds [39].

In recent decades, plant-based proteins have gained increasing attention due to their functional attributes and sustainable production. According to the Food and Agriculture Organization and the World Health Organization, plant-based proteins and foods must be safe and healthy, provide sufficient nutrients, and have a lower environmental impact. To achieve the taste of traditional and animal-based proteins and products, the proper methodology, processing, formulations, and proportions of plant-based foods and proteins should be optimized [44]. Various plant sources, including legumes, cereals, grains, nuts, seeds, seed meals, algal proteins, and mycoproteins, can be utilized for the sustainable production of plant-based proteins and their applications in food. For example, previous researchers have used plant-based protein sources such as wheat gluten, soybeans, peas, peanuts, mung beans, and fava beans as meat analogs [12, 46]. There are several plant-based ingredients, such as proteins, carbohydrates, lipids, mycoproteins, cultured meat, milk & beverages, plant-based cheese, egg analogs, fish alternatives, microalgae, and others (color, flavor, preservatives, cross, etc.), that can be used as an alternative to animal-based proteins and products [41].

**Fig. 3:**
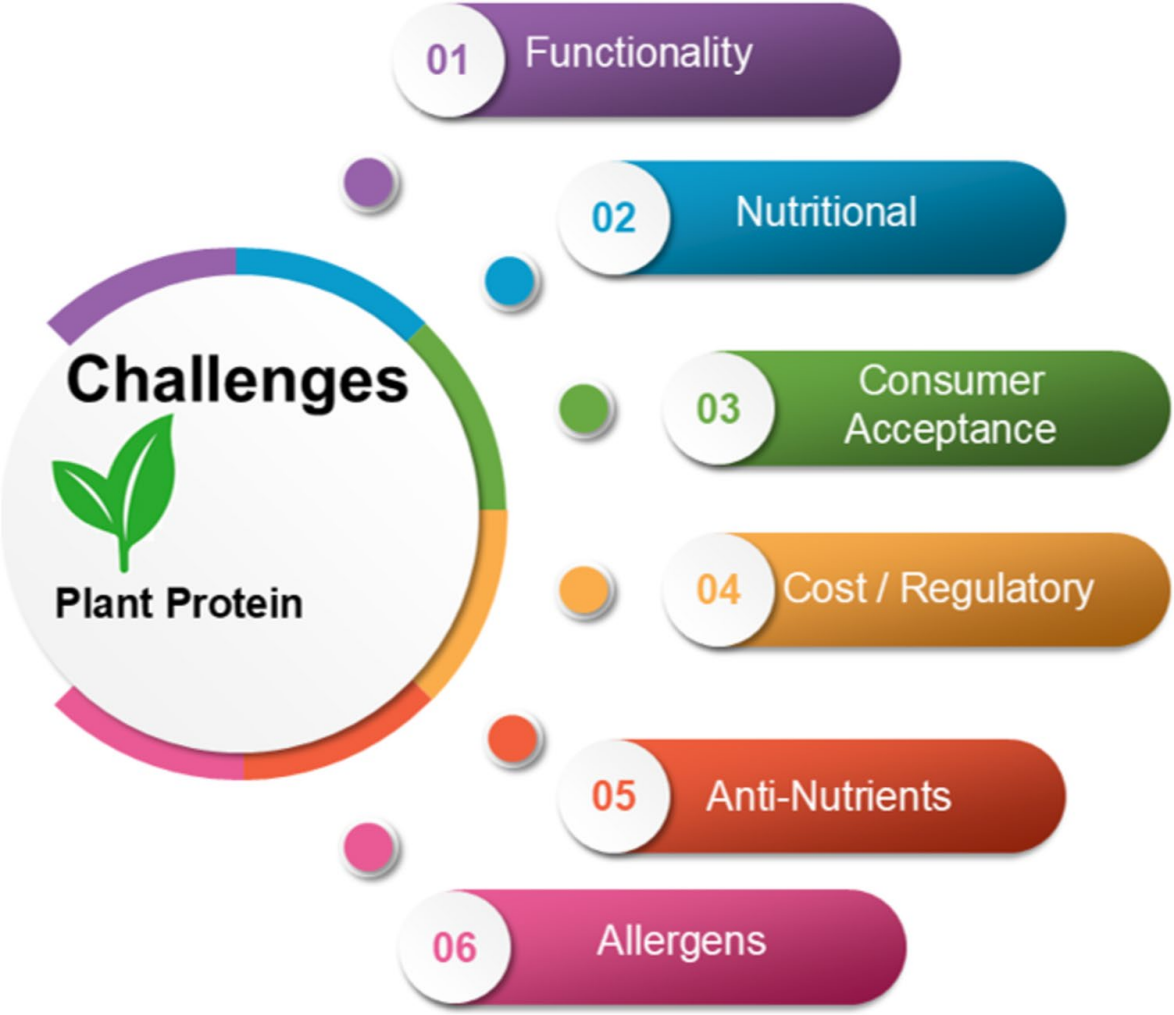
Schematic presentation of challenges of plant proteins in food applications

The rapid increasing demand of the sustainable proteins as an alternative of animal proteins and shifting of the consumers is a crucial challenging step to managing the food safety of alternative proteins source to develop trust in emerging food processing technologies and their validation, maintenance of consumer health, food safety & quality management, nutritional analysis, bioavailability, allergenicity, regulatory and standards, supply chain management and production technology at commercial scale (Fig. 3). A previous study by Hussain and Li [29] highlighted various challenges, including perception and consumer acceptance, food safety issues, allergenicity, regulatory hurdles, and other technological challenges, for plant-based proteins and their food applications.

### Consumer Perceptions

Consumer perception and acceptance of plant-based proteins and foods are significant steps toward scaling food products based on sensory feedback, including texture, appearance, taste, mouthfeel, product origin, food safety, and quality, among others [58]. Several challenges, such as consumer acceptability and awareness, are major hurdles to replacing animal proteins with plant proteins. Various researchers have reported challenges in the acceptance of plant proteins and food products as alternatives to animal proteins. Ettinger et al. [18] investigated consumer acceptability and perception (*n* = 105) of plant-based food products as alternatives to chicken. They reported that most consumers preferred chicken over plant-based alternatives because of its sensory properties.

Therefore, while plant-based proteins and foods are eco-friendly and sustainable sources of nutrients, improving their sensory acceptability remains a significant challenge before they can effectively substitute for animal-based proteins in the market. Moreover, Stubelj et al. [68] investigated the effects of various factors on consumer acceptability of protein sources through a consumer survey study (*n* = 458). His findings suggested that consumer awareness is the most crucial factor in the adoption of plant-based protein sources over animal protein sources. They highlighted that educated consumers intend to substitute animal proteins with plant-based proteins. Furthermore, the lack of education, transparent communication, awareness, cultural sensitivity, and gender-specific approaches are major factors that have affected consumer perceptions of using plant-based proteins as substitutes for animal proteins and foods. On the contrary, the plant (oat, coconut, soy, almond, pea) based dairy alternatives (beverage and cheese) were recommended as substitutes for animal dairy-based beverages [3]. The sensory evaluation study was conducted in two trial steps for plant-based beverages and plant-based cheese, involving 104 and 109 consumers, respectively.

### Risk of Allergenicity and Food Safety Challenges

The presence of allergens and toxins in plant proteins poses significant health and food safety concerns. These substances are linked to diseases such as asthma, eczema, vomiting, breathing issues, and others [40]. The Food and Agriculture Organization and the World Health Organization [27], [34] identified nine major plant-based sources-including tree nuts, wheat, peanuts, sesame, and soybeans—that contain allergens and anti-nutritional factors affecting protein quality. Future research should focus on identifying plant allergens and developing effective reduction strategies to address these health challenges.

The numerous allergen families, such as prolamin, cupin, vicilin, and legumin, as well as pollen allergens and Bet v 1, are derived from various plant sources. The database of the WHO/IUIS Allergen Nomenclature Subcommittee comprises 248 allergen types derived from 76 plant species [79]. Table 2 depicts the common types of protein allergens defined by the European Union [5]. Allergens exhibit diverse properties. Many remain stable during food thermal processing, indicating their resistance to human digestive enzymes. Plant-derived protein allergens can be classified into specific groups, including proteins involved in biotic and abiotic stress responses, protease inhibitors, proteins with structural and biological functions, and entire protein families. For example, plant-based proteins are closely associated with the cupin and prolamin superfamilies, which play an important role in plant defense mechanisms and are often implicated in allergenic responses [9]. These protein groups include well-known allergenic fractions, such as seed storage proteins, which are characterized by high structural stability and resistance to thermal processing and enzymatic digestion. Such properties of proteins may increase their potential to trigger immune reactions. To detect allergens in plant-derived proteins and food products, a variety of advanced analytical and molecular techniques are employed. Commonly used methods include enzyme-linked immunosorbent assay (ELISA), which enables sensitive and specific quantification of allergenic proteins, and polymerase chain reaction (PCR), which allows the identification of allergen-encoding DNA sequences. Additionally, techniques such as flow cytometry, aptamer-based assays, and chromatographic methods (e.g., high-performance liquid chromatography) provide complementary approaches for allergen detection, characterization, and validation. These methods differ in sensitivity and are often used in combination to ensure accurate monitoring of allergen presence in complex food matrices [5].

**Table 2:** Examples of common allergens and allergic proteins defined by the European Union

Plant sources	Allergens	Allergenic proteins
Soybean	Gly m 3/m 5/m 6/m 7/m 8	Profilin, β-conglycinin, glycinin, seed biotinylated protein, 2S albumin
Nuts	Cor a2, Jug r 7, Ana o2, Cor a9, Jug r 4, Pis v2, Ana o1, Cor a11, Jug r 2, Pis v3, Ana o3, Cor a14, Jug r1, Pis v1	Profilin, 11S globulin, 7S vicilin, 2S albumin
Peanuts	Ara h 1/h 2/h 3/h5/h 6/h 7/h 8/h 10/h 11/h 12/h 13/h 14/h 15/h 16/h 17	7S vicilin, 2S albumin, 11S globulin, profilin, lipid transfer protein, oleosin
Sesame	Ses i 1/Ses i 2/Ses i 3/Ses i 4/Ses i 5/Ses i 6/Ses i 7/	2S albumin, 7S vicilin, oleosin, 11S globulin
Wheat	Tri a 37/Tri a 14/Tri a 25/Tri a 19/Tri a 20	αS2 purothionin, lipid transfer protein, thioredoxin, gliadins

To ensure food safety and quality management, it is essential to implement appropriate management and preventive measures. Sabaghi et al. [62] described a range of conventional food processing methods, both thermal and non-thermal (moist heat, dry heat, fermentation, ultrafiltration, proteolysis, high pressure, dehulling) and innovative methods such as microwaves, dielectric heating, ohmic heating, hyperbaric heating, pulsed electric field, cold plasma, ultrasound, magnetic stirring, and ultraviolet radiation. Therefore, maintaining a high level of food safety must be based on a wide range of processing methods that optimize production processes and protect products.

### Regulatory and Technological Challenges

The food industry faces a significant technical challenge in developing plant-based foods that contain complete proteins. Plant-based food products often fail to replicate the attributes of animal-based products, facing several technological hurdles that can hinder sensory, functional, nutritional, and consumer acceptance [39]. A significant limitation is due to poor functional properties, such as water holding, oil holding, and solubility, which affect the sensory attributes, specifically texture, taste, and flavor, of plant-based products. Plant protein-based products often lack the chewy, juicy, and fibrous structure that can enhance their mouthfeel [69]. Plant-based products typically have a beany flavor, an astringent smell, and an off-odor, which contribute to low consumer acceptance. Antinutrients such as saponins, lectins, oxalates, and goitrogens can interfere with the digestibility and absorption of nutrients and micronutrients [1]. Cost-effective scale-up manufacturing is a significant challenge for the food industry. Clear regulations, labeling, and standards for plant protein use should help address the challenges associated with its use in food applications.

Plant proteins have GRAS status in the USA. The guidelines and regulations for novel foods and plant proteins are very limited worldwide. The researchers' focus should be on the international standard for novel foods and plant-derived protein alternatives to make them suitable for food applications. Countries such as Singapore, Thailand, and South Korea are the only ones with standard guidelines [31]. Fermentation and genetically modified methods are the two emerging approaches to producing plant-based foods that mimic the original animal-derived products. Additionally, there are no regulatory guidelines for labeling such a product. Only China, South Korea, Singapore, and Thailand have specific regulations for novel foods [39]. The lack of clear, transparent regulatory guidelines for alternative proteins hampers the food industry's progress. Moreover, terms such as vegetarian sausage and vegan bacon on the packaging of plant-based meat have been banned in France, which can mislead consumers. Additionally, plant-based meat substitutes made from peas, soy, and legumes are not permitted to be labeled as steak, burger, fillet, sausage, chicken, etc. Several states in the United States have restrictive regulations that prohibit meat alternatives from being as similar to conventional meat as they are used [66].

## Conclusions

Plant-based proteins are a key area of focus in food science and biotechnology, offering a sustainable pathway to meet nutritional demands. Various natural sources, such as cereals, nuts, oilseeds, peel, kernels, and other plant sources, can be used to produce eco-friendly protein alternatives using traditional and emerging extraction technologies. The emerging technologies, including PEF, ultrasound, supercritical fluid extraction, microwaves, high pressure, and enzymatic methods, can isolate proteins from plant sources with higher yields and better quality than traditional methods. The successful integration of these proteins into the food matrix depends not only on extraction efficiency but also on overcoming deep-seated challenges in allergenicity, food safety, and regulatory alignment. Such protein sources can serve as alternatives to animal proteins to develop various food products (meat analogs, dairy alternatives, baked products, snack products, and fortified beverages) and to address food processing challenges. Moreover, technological challenges are the major factors inhibiting the scaling up of plant-based proteins, as is consumer awareness. The extraction and processing technologies, whether combined or not, may focus on improving functionality, optimizing nutritional profiles, enhancing amino acid composition, and addressing sensory challenges associated with plant-based proteins for food applications in the future.

## Data Availability

Not applicable.
